# Cellulose Acetate/Hydroxyapatite-Dexamethasone Loaded Membranes for the Prevention of Implant-Associated Acute Inflammation

**DOI:** 10.3390/polym18101159

**Published:** 2026-05-08

**Authors:** Stefan Ioan Voicu, Andreea Madalina Pandele, Adrian Ionut Nicoara, Iulian Vasile Antoniac, Madalina Oprea, Cristian Bica

**Affiliations:** 1 Department of Analytical Chemistry and Environmental Engineering, Faculty of Chemical Engineering and Biotechnologies, National University of Science and Technology POLITEHNICA Bucharest, 1-7 Gheorghe Polizu, 011061 Bucharest, Romania; 2 Advanced Polymers Materials Group, National University of Science and Technology POLITEHNICA Bucharest, 1-7 Gheorghe Polizu, 011061 Bucharest, Romania; 3 Academy of Romanian Scientists, 54 Splaiul Independentei Street, District 5, 050094 Bucharest, Romania; 4 Department of Science and Engineering of Oxide Materials and Nanomaterials, Faculty of Chemical Engineering and Biotechnologies, National University of Science and Technology POLITEHNICA Bucharest, 1-7 Gheorghe Polizu, 011061 Bucharest, Romania; 5 Faculty of Materials Science and Engineering, National University of Science and Technology POLITEHNICA Bucharest, 313 Splaiul Independentei, 060042 Bucharest, Romania; 6 Department of Orthopedy and Traumatology, Faculty of Medicine and Farmacy Craiova, Petru Rares 2, 200349 Craiova, Romania; 7 NeuroHope Hospital, 13 Pechea, 013982 Bucharest, Romania

**Keywords:** cellulose acetate, hydroxyapatite, dexamethasone, composite membrane, implant-associated inflammation, biomineralization, controlled drug release

## Abstract

Implant-associated acute inflammation remains a major challenge in orthopedic, dental, and maxillofacial applications, often impairing osseointegration and leading to early implant failure. In this study, multifunctional cellulose acetate/hydroxyapatite–dexamethasone (CA/HA–DEXA) composite membranes were developed to locally modulate inflammation while supporting early bone–implant interactions. Cellulose acetate provides a flexible matrix, hydroxyapatite enhances bioactivity and osteoconductivity, and dexamethasone acts as an anti-inflammatory agent. The membranes exhibited composition-dependent swelling and drug release behavior. The swelling degree decreased from ~11% for pristine CA to ~5% for the highest HA–DEXA loading, indicating a denser structure with restricted water uptake. Dexamethasone release showed a biphasic profile, with cumulative release reaching ~68%, ~88%, and ~93% for 0.5%, 1%, and 2% HA–DEXA loadings after 72 h, respectively. In vitro evaluation indicated improved biomineralization for CA/HA–DEXA membranes compared to neat CA, attributed to the role of hydroxyapatite as a nucleation promoter. These findings suggest that CA/HA–DEXA composite membranes represent a promising strategy for controlling early inflammatory responses while supporting bone regeneration at the implant interface.

## 1. Introduction

Implant-associated acute inflammation is considered an important limitation in orthopedic, dental, and maxillofacial applications, because it often leads to reduced osseointegration, delayed healing, and early implant failure [1]. The initial inflammatory response to biomaterial-based implants is initiated by surgical trauma and foreign body reactions, and it is characterized by macrophage activation and excessive production of pro-inflammatory markers such as tumor necrosis factor-α (TNF-α), interleukin-1β (IL-1β), and interleukin-6 (IL-6) [2]. Even if the initial inflammatory phase is necessary for tissue regeneration, uncontrolled or prolonged inflammation leads to fibrous encapsulation and compromises the long-term stability of the implant [3]. Therefore, the development of biomaterials capable of locally modulating the inflammatory response and maintaining mechanical integrity and bioactivity has started gaining increasing research attention lately [4].

Cellulose acetate (CA) is a renewable, semi-synthetic polymer extensively used for biomedical applications due to its biocompatibility, film-forming ability, chemical stability, and tunable surface properties [5]. Previous studies demonstrated that CA is an excellent candidate for biomedical membrane fabrication, drug delivery systems, and tissue engineering scaffolds with favorable porosity and mechanical properties [6]. Moreover, by tuning the polymer’s degree of substitution, hydrophilicity, degradation, and diffusional characteristics can be controlled, thus making CA particularly attractive for controlled release platforms [7]. Still, neat CA does not possess intrinsic bioactivity and osteoconductivity properties, which limit its performance in bone-related applications [8].

Hydroxyapatite (HA) is a calcium phosphate bio-ceramic that represents the main mineral component of native bones [9]. Throughout time, it was widely incorporated into polymeric matrices to enhance bioactivity and interfacial bonding with host tissue [10]. Numerous studies have shown that HA-containing composites improve protein adsorption, promote osteoblast adhesion, and stimulate osteogenic differentiation [11,12]. Furthermore, CA/HA composite systems were previously explored, mainly focusing on mechanical reinforcement and osteoconductive performance [13]. However, to the best of our knowledge, existing CA/HA-based composites remain passive and do not actively regulate the peri-implant inflammatory microenvironment.

Dexamethasone (DEXA) is a potent synthetic glucocorticoid widely applied in clinical practice for the management of acute inflammation. Its mechanism of action involves inhibition of nuclear factor-κB (NF-κB) signaling and downregulation of inflammatory gene expression [14,15]. Despite its efficacy, systemic DEXA administration is associated with adverse effects such as delayed wound healing, immunosuppression, and metabolic dysregulation [16]. To overcome these limitations, targeted DEXA delivery systems have been developed using polymeric carriers and coatings [17], and nanoparticulate systems [18,19] to achieve controlled, site-specific drug release. From a biological perspective, the incorporation of dexamethasone into polymeric membranes designed for implant coating could provide an active strategy to modulate early inflammatory cascades at the implant interface. The composite membrane may facilitate a transition to a pro-regenerative microenvironment by locally inhibiting NF-κB signaling and lowering the production of pro-inflammatory cytokines [20]. This localized strategy addresses uncontrolled acute inflammation, one of the main causes of early implant failure, while minimizing systemic exposure [21]. Nonetheless, research continues to focus on attaining a reliable and consistent delivery profile while maintaining material functionality and cytocompatibility [19].

In this context, multifunctional composite membranes containing cellulose acetate, hydroxyapatite, and dexamethasone could be an effective strategy to address both structural and biological requirements of implantable biomaterials. In the proposed design, CA acts as a flexible polymeric matrix that supports bone tissue formation around the implant. HA is used as a drug loading agent to minimize burst release and provides bioactivity, mechanical reinforcement, and osteoconductive properties. DEXA acts as an active immunomodulatory agent that locally suppresses excessive inflammatory responses and promotes a pro-regenerative microenvironment.

The novelty of this study lies in the development of a single-platform composite membrane (CA/HA-DEXA) that simultaneously integrates mechanical support, osteoconductive bioactivity, and controlled delivery of anti-inflammatory drugs—functionalities that have previously been addressed only separately. Unlike conventional CA-based drug delivery systems, which lack bioactive reinforcement [22], or HA-reinforced composites, which do not provide pharmacological action [23], and DEXA-loaded coatings, which are usually surface-based and prone to burst release [24], the proposed system incorporates both HA and DEXA homogeneously into the cellulose acetate matrix. This integrated architecture enables synergistic performance, combining structural stability with sustained drug release targeted to inflammation. In addition, an optimized manufacturing route is introduced to ensure uniform HA/DEXA dispersion, minimizing burst release and enhancing long-term therapeutic efficacy—an aspect insufficiently addressed in previous studies.

## 2. Materials and Methods

This study describes the synthesis of cellulose acetate/hydroxyapatite-dexamethasone composite membranes with anti-inflammatory and pro-biomineralization properties. Composite membrane synthesis can be achieved through two primary techniques: solution casting with subsequent solvent evaporation and phase inversion. However, in the case of cellulose acetate, the most suitable membrane preparation technique is phase inversion, as research shows that CA-membranes obtained by solvent evaporation may have a higher friability. Moreover, this method also prevents potential clustering of the filler at the base of the membrane, which can happen during solvent evaporation [25]. Detailed information about the materials used, HA-loading with the anti-inflammatory agent and membrane preparation procedure is provided in the sections below.

### 2.1. Chemicals and Reagents

Cellulose acetate powder (average Mn ~30,000, determined by GPC) was procured from Sigma-Aldrich (Burlington, MA, USA). N,N-dimethylformamide (DMF, 99%, Honeywell, Charlotte, NC, USA) was used as the solvent for polymer dissolution. Hydroxyapatite particles were derived from spongy bone samples following a method previously developed by our research group [26]. Dexamethasone injectable solution (4 mg/mL), supplied by Rompharm (Otopeni, Romania), served as the anti-inflammatory agent.

For mineralization studies, calcium chloride (94%, Roth, Karlsruhe, Germany), hydrochloric acid (37%, Sigma-Aldrich, Burlington, MA, USA), tris(hydroxymethyl)aminomethane (99.8%, Sigma-Aldrich, Burlington, MA, USA), and anhydrous disodium phosphate (99%, Sigma-Aldrich, Burlington, MA, USA) were employed. All chemicals were of analytical grade and used as received without further purification. Distilled water was used throughout all experimental procedures.

### 2.2. Synthesis of the Composite CA/HA-DEXA Membranes

In the first stage, hydroxyapatite particles were loaded with dexamethasone (HA-DEXA). Specifically, 0.5 g of hydroxyapatite and 2 mL of dexamethasone solution were dispersed in 10 mL of DMF and homogenized under magnetic stirring for 2 h at 45 °C. The resulting mixture was subsequently dried in a vacuum oven at 40 °C for 72 h. The dried material was collected and finely ground using a mortar and pestle prior to further use.

**Figure 1:**
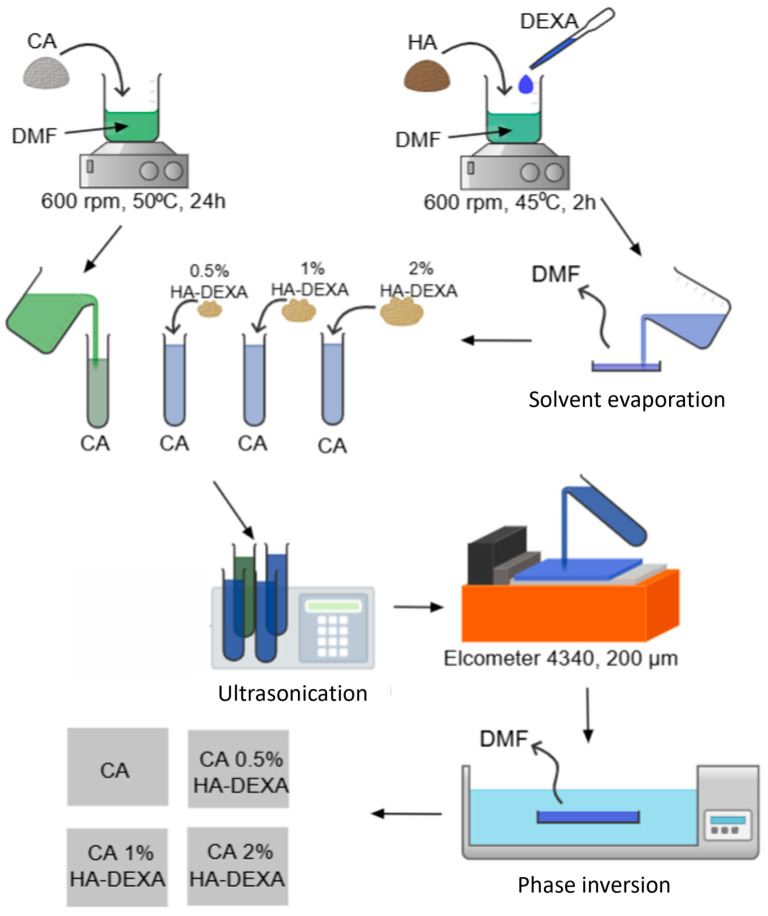
Technological workflow was followed for the synthesis of the composite CA/HA-DEXA membranes.

The technological workflow followed for membrane synthesis was graphically represented in Figure 1. Neat and composite membranes were prepared starting from a 12 wt% cellulose acetate solution. For composite samples, different amounts of HA-DEXA filler (0.5, 1, and 2 wt% reported to the polymer mass) were incorporated. Cellulose acetate (12 g) was dissolved in 176 mL of DMF under magnetic stirring at 600 rpm for 6 h at 40 °C. Composite membranes were fabricated following a method previously developed by our research group [27]. The required amount of HA-DEXA powder was added to the polymer solution and stirred magnetically until complete homogenization (approximately 6 h at 700 rpm). To eliminate possible agglomerates and ensure uniform dispersion of the filler, the resulting suspensions were further subjected to ultrasonic treatment for 30 min using an ultrasonic processor (UP100H, 80% amplitude).

In the final step, the polymer solutions were cast onto a glass plate using an automatic film applicator (Elcometer 4340, Elcometer, Warren, MI, USA), followed by gentle immersion in a coagulation bath containing 500 mL of distilled water at 25 °C. The resulting membranes (10 × 10 cm surface area, approximately 200 μm thickness) were then removed from the bath, thoroughly washed with ethanol and distilled water to eliminate residual DMF, and subsequently dried in a vacuum oven at 40 °C for 24 h.

The obtained membranes were further subjected to physicochemical characterization by ATR-FTIR, XPS, SEM, and XRD analyses. In addition, their biomineralization capacity was evaluated using the Taguchi method.

### 2.3. Biomineralization Study

After complete drying, the membranes were subjected to in vitro mineralization to evaluate the formation of an inorganic phase on their surface under simulated physiological conditions. Biomineralization was performed using the alternate soaking method described by Taguchi et al. and previously applied in our research group [28].

Briefly, the samples were first immersed in a 200 mM CaCl_2_ solution at 37 °C for 24 h, with the pH adjusted to 7.4 using HCl and Tris base. The membranes were then rinsed with distilled water and subsequently incubated in a 120 mM Na_2_HPO_4_ solution at 37 °C for an additional 24 h. This cycle was repeated twice. Finally, the membranes were thoroughly washed with distilled water and dried at 37 °C for 72 h prior to characterization.

### 2.4. Characterization Methods

ATR-FTIR spectra were recorded using a Bruker VERTEX 70 spectrometer (Bruker, Billerica, MA, USA) equipped with a diamond ATR accessory, in the 4000–600 cm^−1^ range, at a resolution of 4 cm^−1^, averaging 32 scans per sample.

Surface chemistry was analyzed by X-ray Photoelectron Spectroscopy (XPS) using a K-Alpha instrument (Thermo Fisher Scientific, Waltham, MA, USA) with a monochromated Al Kα source (1486.6 eV) under a base pressure of 2 × 10^−9^ mbar. Charge compensation was achieved using a flood gun, and binding energies were calibrated to the C 1s peak at 284.4 eV. Survey and high-resolution spectra were acquired at pass energies of 200 eV and 20 eV, respectively, and peak deconvolution (C 1s, O 1s, N 1s) was performed using Gaussian–Lorentzian functions.

Morphology was examined by scanning electron microscopy (SEM) using a Quanta Inspect F microscope (Hillsboro, OR, USA) equipped with an EDX detector, at an accelerating voltage of 30 kV.

Phase composition and crystallinity were investigated by X-ray diffraction (XRD) using a Shimadzu XRD 6000 (Shimadzu, Kyoto, Japan) with Cu Kα radiation (λ = 1.54 Å), Ni-filtered, over a 2θ range of 0–80°.

Swelling behavior was determined by immersing 1 cm^2^ dry membrane samples in distilled water at room temperature, and the swelling degree was calculated using the following equation:

$$Swelling\left[\%\right]=\frac{Ws-Wd}{Ws}\times 100$$

where Ws and Wd denote the weights of the swollen and dry membranes, respectively. Measurements were performed at predetermined time intervals to monitor swelling kinetics.

Dexamethasone release was assessed by UV-Vis spectrophotometry (Shimadzu UV-3600). Membrane samples (2 × 2 cm, 26 ± 2 mg) were placed in dialysis bags containing 4 mL PBS, then immersed in 200 mL PBS and agitated at 100 rpm for 72 h at room temperature. Absorbance was measured at 237 nm, using a calibration curve (2–10 μg/mL). Sink conditions were maintained throughout the experiment by using a large volume of release medium (200 mL PBS) relative to the amount of loaded drug, ensuring that the dexamethasone concentration remained well below its solubility limit. Continuous agitation (100 rpm) further supported uniform distribution and minimized concentration gradients.

The swelling degree and drug release results are expressed as the mean of three independent measurements ± standard deviation (mean ± SD), with standard deviation calculated using Microsoft Excel.

## 3. Results

The present study systematically investigates the physicochemical and in vitro biomineralization properties of CA/HA–DEXA membranes, along with their swelling and drug release behavior. The investigation of these features is particularly significant for determining whether the developed membranes could be promising candidates for next-generation polymeric biomaterials in implant-related applications.

### 3.1. ATR FT-IR

**Figure 2:**
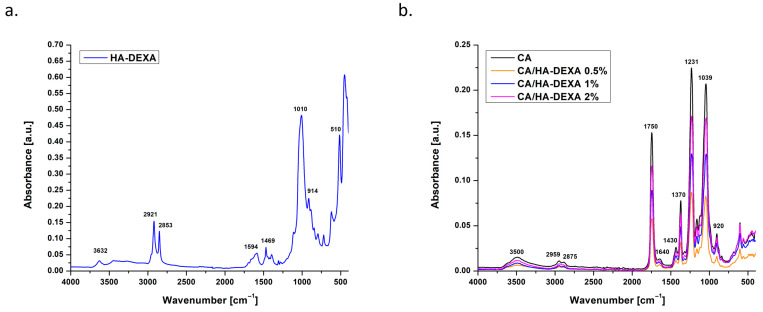
ATR FT-IR spectra of HA powder loaded with DEXA (**a**) and CA-based membranes (**b**).

The ATR FT-IR analysis of the HA–DEXA powder (Figure 2a) confirmed that dexamethasone was not thermally or chemically degraded during the loading process and was stably incorporated in the HA particles, peaks specific to both components being observed in the recorded spectrum. The peaks located at 1010, 914 and 510 cm^−1^ were attributed to the stretching vibrations of the phosphate groups (PO_4_^3−^), while the one at 630 cm^−1^ was generated by the bending vibrations specific to the crystalline structure of HA [29]. Regarding the organic component, the peaks at 2921 and 2853 cm^−1^ correspond to the stretching vibrations of the C–H bonds in the methyl and methylene groups, characteristic of the aliphatic chains in the dexamethasone structure. The signals in the 1594–1469 cm^−1^ region can be associated with the stretching vibrations of the aromatic-condensed rings in the glucocorticoid structure, while the band at 1250 cm^−1^ can be attributed to ether bonds (C–O–C) and possible ester groups, characteristic of the steroid nucleus [30,31].

The spectrum of the neat CA membrane (Figure 2b) was dominated by clear and intense peaks, specific to the polymer structure and in good agreement with previously reported results [32]. The spectral attribution of each peak was detailed in Table 1. After the addition of HA-DEXA, progressive and relevant changes were observed. The 0.5% CA/HA-DEXA sample showed a slight decrease in the intensity of the bands characteristic of esters (1750 cm^−1^) and a broadening of the signal in the 1000–1100 cm^−1^ area, suggesting a potential interaction between the inorganic phase and the polymer matrix. Also, in this sample, the peak located at 920 cm^−1^ was outlined for the first time, weakly visible, but specific to the stretching vibrations of the phosphate groups in HA. As the filler concentration increased (CA/HA-DEXA 1% and CA/HA-DEXA 2%), the signal at 920 cm^−1^ was accentuated, which suggested a higher effective loading with the inorganic phase. This evolution was also supported by a slight change in the shape of the peaks in the 1000–1100 cm^−1^ region (1039 cm^−1^ in particular), which undergo an overlap with the bands originating from HA. At 2% HA-DEXA loading, the relative intensity of the characteristic CA bands decreased slightly and the signal at 1430 cm^−1^ became weaker and less defined, which may indicate a subtle modification of the local structural order in the polymer chain, possibly due to the intercalation of HA particles [33].

Considering the fact that the membrane with 2% HA–DEXA loading presented the most evident indications of filler incorporation into the polymer matrix, only this sample and the neat cellulose acetate membrane were selected for further analysis.

### 3.2. XPS

X-ray photoelectron spectroscopy was employed to investigate the surface chemical composition of the samples. Survey spectra as well as high-resolution C 1s and O 1s spectra were recorded to evaluate the influence of HA-DEXA incorporation on the membrane surface chemistry.

**Figure 3:**
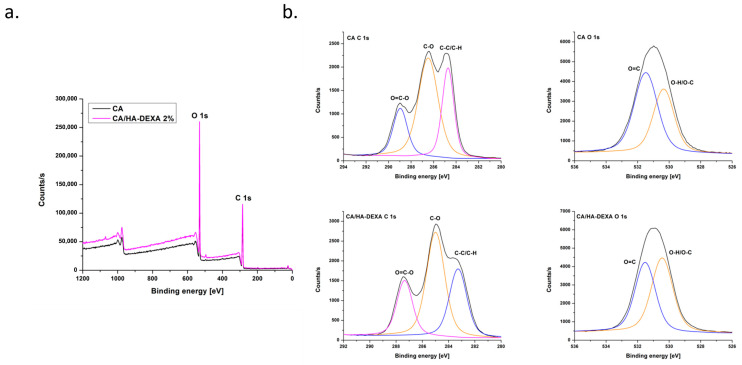
Survey (**a**) and high resolution (**b**) XPS spectra of CA and CA/HA-DEXA 2% membranes.

The survey XPS spectra (Figure 3a) of both samples were dominated by intense C 1s and O 1s signals, indicating that the major surface components are carbon and oxygen-containing functional groups. No additional peaks were detected in the CA/HA-DEXA spectrum, most likely due to the embedding of HA-DEXA within the polymer matrix or due to the low amount of filler reported to the polymer mass.

After deconvolution, the high-resolution C 1s spectra of neat CA showed the presence of three subpeaks located at 284.8, 286.5 and 288.9 eV, corresponding to C-C/C-H, C-O bonds from the cellulose backbone and O-C=O groups specific to the ester moieties of CA, respectively [34]. No significant changes were observed in the C 1s spectrum for the CA/HA–DEXA membrane, indicating that the surface chemistry of CA was maintained after filler incorporation. However, slight differences in the relative intensities of the C–O and O–C=O peaks can be noted, which may relate to the contribution of oxygen-rich functional groups from HA-DEXA. The lack of any additional peaks related to carbon could suggest that dexamethasone was distributed within the CA matrix instead of creating a separate surface layer.

The O 1s spectrum of neat CA displayed two peaks at 530.4 and 531.5 eV, attributed to O–H and O-C groups and carbonyl oxygen (O=C), respectively. This spectral profile is characteristic of cellulose acetate and reflects the coexistence of ester and hydroxyl functionalities at the membrane surface [34]. Similarly to the C 1s spectrum, the O 1s spectrum of the CA/HA–DEXA membrane presents only subtle changes in the peak intensity distribution, the contribution of O–H/O-C being increased, most likely due to the presence of additional hydroxyl groups from hydroxyapatite and dexamethasone.

The XPS analysis indicated that the addition of HA-DEXA does not substantially modify the surface chemistry of the membranes. However, the slight differences observed in the C 1s and O 1s components suggest that the filler was successfully integrated into the membrane structure, without altering the surface chemical structure of CA.

### 3.3. Biomineralization Study

#### 3.3.1. SEM

**Figure 4:**
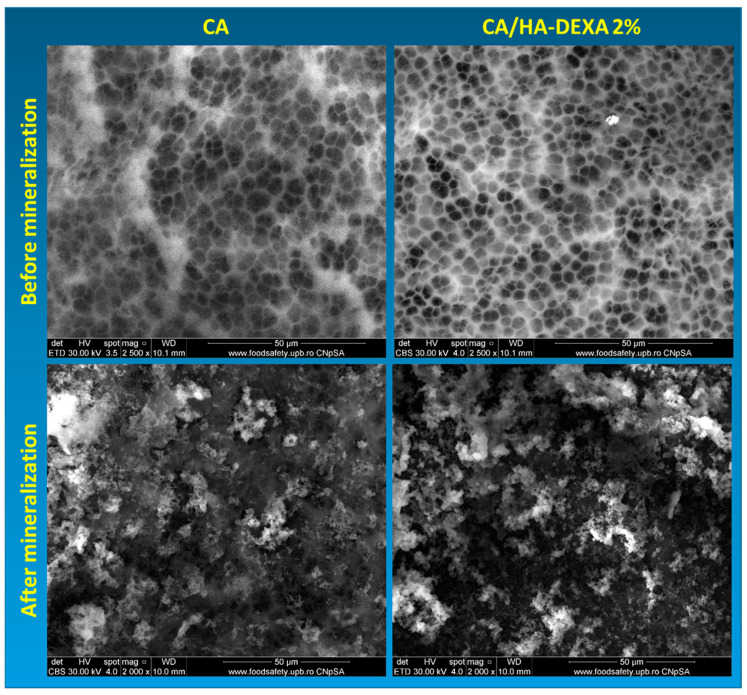
SEM images of CA and CA/HA-DEXA 2% membranes before and after Taguchi mineralization.

The qualitative analysis of the surface morphology of neat cellulose acetate membranes and CA membranes loaded with HA-DEXA was performed via SEM before and after the Taguchi biomineralization procedure (Figure 4). Representative micrographs were recorded at 2500× magnification for the non-biomineralized membranes and at 2000× magnification after biomineralization, with a scale bar of 50 μm.

Prior to biomineralization, the neat CA membrane exhibited a porous morphology with interconnected pores and relatively smooth pore walls, typical of phase-inverted cellulose acetate membranes. The pore size distribution was uniform, indicating a homogeneous membrane formation process [35]. The non-biomineralized CA/HA–DEXA membrane showed subtle morphological changes compared to neat CA. The incorporation of HA-DEXA within the polymer matrix led to a more heterogeneous surface, characterized by increased roughness and a broader pore size distribution. Irregular pore contours and locally denser regions were observed and were attributed to the influence of the inorganic filler particles in the solvent–nonsolvent exchange during the phase inversion process. No continuous inorganic surface layer was noticed, thus confirming that HA was embedded or just partially exposed within the polymer matrix, as it was also indicated by XPS.

Following the Taguchi biomineralization, significant changes in the surface morphology of both neat and composite membranes were observed. The surfaces became partially covered by mineral deposits, forming irregular aggregates distributed across the membrane surface and along the pore walls. Despite this surface mineral deposition, the underlying porous architecture remained visible, indicating that biomineralization occurred without complete pore blockage.

Notably, the CA/HA–DEXA membrane displays a more pronounced and homogeneous mineral coverage compared to neat CA. This observation suggests that the pre-loaded HA-DEXA facilitated calcium phosphate crystals nucleation and growth, leading to enhanced surface mineralization [36]. The increased density of mineral deposits on the composite membrane further highlights the role of hydroxyapatite as an effective nucleation promoter, while preserving the structural integrity of the porous membrane.

#### 3.3.2. XRD

**Figure 5:**
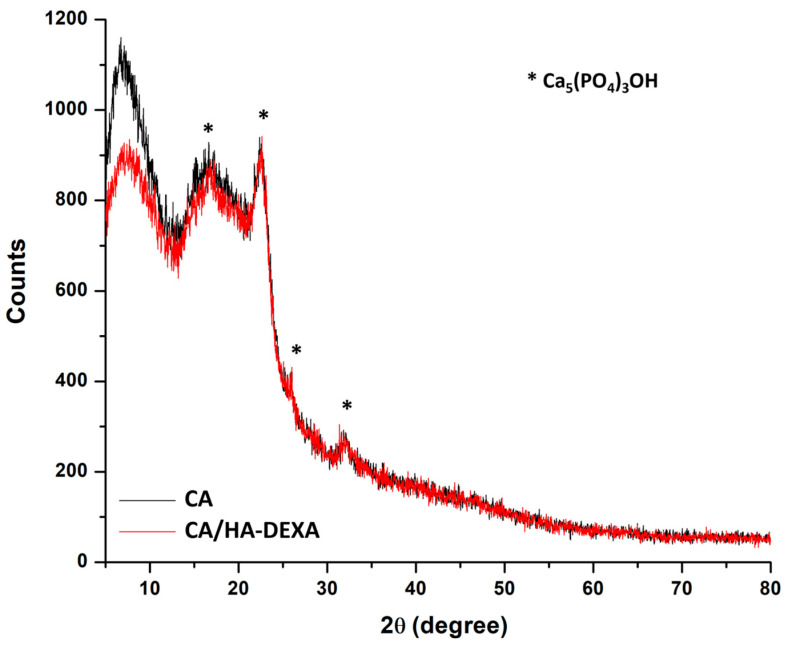
XRD patterns obtained for the CA and CA/HA-DEXA 2% membranes after Taguchi mineralization.

X-ray diffraction (XRD) analysis was performed to investigate the structural characteristics of the membranes after Taguchi biomineralization. The diffractograms were recorded over a 2θ range of 5–80° using Cu Kα radiation (Figure 5). The XRD pattern of the biomineralized cellulose acetate (CA) membrane exhibited a broad diffraction halo in the low-angle region (2θ ≈ 8–25°). The peaks characteristic of cellulose acetate were identified at 2θ values of approximately 7.16°, 16.78°, and 22.59°, and due to their aspect were correlated with a predominantly amorphous structure [37,38]. At higher angles (2θ ≈ 18–35°), low-intensity peaks corresponding to hydroxyapatite (Ca_5_(PO_4_)_3_OH) were observed, suggesting the formation of calcium phosphate phases during biomineralization [28]. The low intensity and broadness of these peaks indicated limited crystalline content and/or poorly ordered domains.

The CA/HA–DEXA composite membrane displayed a similar amorphous halo, indicating that the incorporation of the inorganic phase does not significantly alter the structural organization of the cellulose acetate matrix. However, a higher number of HA-related peaks can be distinguished, suggesting an increased presence of mineral phases. The relatively low intensity of these peaks, however, suggests that no large crystalline domains were formed, which may indicate a relatively uniform distribution of the inorganic phase within the polymer matrix.

Overall, the XRD results indicate that both membranes are predominantly amorphous, with minor crystalline contributions associated with biomineralized hydroxyapatite. These results were in good agreement with those from previously performed analysis and confirmed the fact that HA-DEXA enhances biomineralization by acting as an effective nucleation promoter for calcium phosphates.

### 3.4. Swelling Degree and Drug Release Study

**Figure 6:**
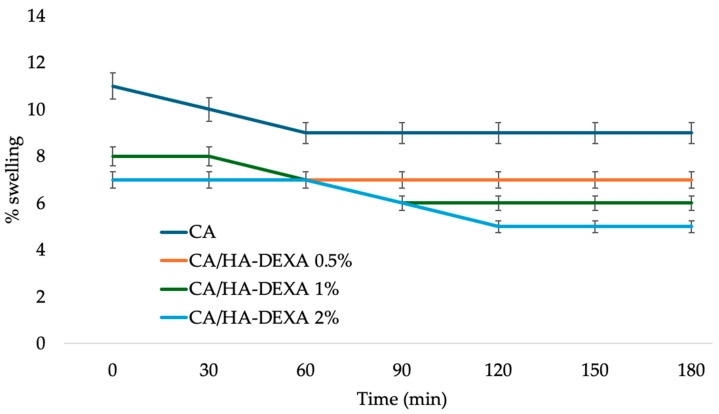
Graphical representation of the swelling behavior of the neat and composite CA-based membranes. Data are presented as mean ± SD (n = 3).

The swelling behavior of the membranes (Figure 6) showed a clear dependence on the incorporation and loading of HA–DEXA filler. The pristine cellulose acetate membrane exhibited the highest swelling degree (~11% initially), which gradually decreased and stabilized around 9% after 60 min, indicating rapid water uptake followed by equilibrium swelling [39]. In contrast, all composite membranes display reduced swelling, with the extent of reduction increasing proportionally to the HA–DEXA content. The CA/HA–DEXA 0.5% sample maintained a relatively stable swelling degree of ~7%, suggesting a modest restriction of water diffusion due to partial pore occupation. At higher loadings, the effect becomes more pronounced: the 1% composite decreases to ~6%, while the 2% composite reaches the lowest equilibrium swelling (~5%). This behavior can be attributed to the progressive reduction in free volume and polymer chain mobility, as well as enhanced interfacial interactions between cellulose acetate and the inorganic filler, leading to a denser membrane structure and partial pore blockage [40].

**Figure 7:**
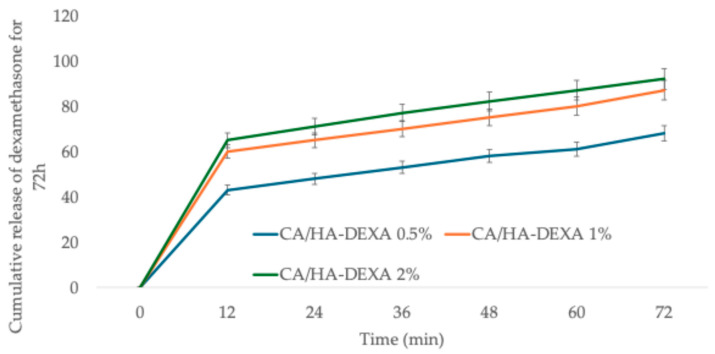
Cumulative release of dexamethasone from the composite CA/HA-DEXA membranes for 72 h. Data are presented as mean ± SD (n = 3).

Figure 7 illustrates the cumulative release of DEXA from the synthesized membranes. The dexamethasone release profiles exhibit a biphasic behavior, with an initial burst release within the first 12 h followed by a sustained, diffusion-controlled stage for up to 72 h. The cumulative release increases with HA–DEXA content, reaching approximately ~68% (0.5%), ~88% (1%), and ~93% (2%), reflecting the higher drug loading at increased filler concentrations. In correlation with the swelling results, although higher HA–DEXA loadings reduce the swelling degree—indicating a denser and more restricted polymer network—the overall drug release is enhanced. This suggests that drug loading plays a dominant role over diffusional limitations. The reduced swelling likely contributes to the sustained release phase by limiting water uptake and polymer relaxation, while the higher concentration gradient at increased loadings drives greater cumulative release [41].

Overall, the results demonstrate that increasing HA–DEXA loading effectively enhances the dimensional stability of the membranes, which is advantageous for applications requiring controlled fluid uptake and sustained drug release. The composite membranes demonstrated tunable release behavior, combining controlled swelling with sustained drug delivery.

## 4. Discussion

Considering the results obtained, it can be stated that the HA-DEXA filler was successfully incorporated within the CA matrix, the absence of additional XPS signals in the composite membranes spectrum indicating that the filler was more likely distributed in the polymer bulk rather than creating a superficial coating layer. According to previous studies, this membrane architecture is particularly effective for controlled drug release because it restricts burst, surface-associated diffusion and promotes a more prolonged release profile [41].

The increased mineral deposition observed in the SEM images and XRD spectrum of CA/HA-DEXA 2% membranes supports hydroxyapatite’s dual function—as a calcium phosphate nucleation enhancer and structural reinforcer for the polymer matrix. This enhanced biomineralization ability of the composite membranes could be explained by the fact that heterogeneous nucleation is facilitated by domains rich in calcium and phosphate, which lowers the activation energy needed for apatite production during the alternative soaking process [42].

Furthermore, the porous architecture was unaffected by the mineral phase formation, suggesting that the composite membranes retain their permeability while developing bioactive properties. This behavior was also observed by Fornazier et al. in the case of solvent-cast cellulose acetate/calcium glycerophosphate membranes [43] and it indicates that CA membranes are appropriate candidates for the improvement of in vitro biomineralization of implants.

The swelling degree obtained for the neat cellulose acetate membrane is in accordance with previously reported results in the literature [44], the decrease in the case of composite membranes being explained by the volume occupied in the membrane structure by HA particles and by the fact that a composite membrane is more stable in comparison with neat polymer. Several systems for the release of dexamethasone have been reported, such as supramolecular hydrogels [45,46,47,48], nanocarriers [49,50] or polymer fibers [51,52,53]. Best release results were reported from gels due to the high diffusivity through the polymer network (90–100% release in 48–72 h), with intermediary results for polymer fibers (75–95% release in 48–72 h), while the nanocarriers can be designed to allow release up to several days.

The swelling behavior reflects the hydrophilic nature of cellulose acetate and its interconnected porous structure. The incorporation of HA–DEXA leads to a decrease in water uptake, likely due to the microstructural heterogeneity. The drug release profile suggests a predominantly diffusion-controlled mechanism, where the polymer matrix acts as a barrier that regulates dexamethasone transport toward the surrounding medium. The preliminary loading of DEXA onto HA particles appears to mitigate burst release, supporting a more sustained therapeutic delivery.

## 5. Conclusions

This study reports the preparation and characterization of cellulose acetate/hydroxyapatite–dexamethasone (CA/HA–DEXA) composite membranes intended for modulation of implant-associated inflammation and support of early biomineralization. The membranes were fabricated via phase inversion and evaluated to assess their suitability for this application.

FT-IR, XPS, and XRD analyses support the incorporation of HA–DEXA within the CA matrix while preserving its semi-amorphous structure, with more evident features at higher loadings (2%). SEM and XRD results indicate increased surface roughness and enhanced biomineralization compared to neat CA, consistent with the role of HA as a nucleation site for calcium phosphate deposition.

Swelling decreased from ~11% (CA) to ~5% (CA/HA–DEXA 2%), suggesting reduced water uptake in the composites. Dexamethasone release showed a biphasic profile, reaching ~68–93% cumulative release over 72 h depending on HA–DEXA content, indicative of a diffusion-influenced process with a contribution from drug loading.

Overall, the results suggest that CA/HA–DEXA membranes may provide a platform for localized drug delivery and improved mineralization at the implant interface. Further studies, particularly cytokine profiling and macrophage response assays, are necessary to confirm immunomodulatory effects and long-term performance.

## Figures and Tables

**Table 1:** Significant ATR FT-IR peaks observed in the CA/HA-DEXA spectra and their spectral attributions.

Wavenumber [cm^−1^]	Type of Bond Vibration	Description
3500	O–H stretching	Hydroxyl groups from CA or traces of water
2959/2875	C–H stretching	Aliphatic bonds –CH_3_ and –CH_2_– in the polymer structure
1750	C=O stretching	Ester group characteristic to CA
1640	H–O–H bending	Physically retained water on the membrane surface
1430	–CH_2_– bending	Aliphatic bonds from the main chain of the polymer
1370	C–H stretching	Methyl groups in the acetate structure
1231	C–O stretching	Ester bonds characteristic to CA
1039	C–O–C stretching	Ether bonds in the polymer network
920	PO_4_^3−^ stretching	Specific signature for hydroxyapatite (HA); increases with dosage

## Data Availability

The original contributions presented in this study are included in the article. Further inquiries can be directed to the corresponding author.
